# Reliability and predictive validity of the bedside upper limb functional evaluation tool (BUFET) among stroke subjects – a psychometric study

**DOI:** 10.1038/s41598-025-28744-6

**Published:** 2025-11-23

**Authors:** Shruti Mohapatra, Abraham M. Joshua, Shreekanth D. Karnad, Shyam Krishnan, Prasanna Mithra, Zulkifli Misri, Shivananda Pai, Rohit Pai

**Affiliations:** 1 Department of Physiotherapy, Kasturba Medical College Mangalore, Manipal Academy of Higher Education, Manipal, India; 2 Department of Community Medicine, Kasturba Medical College Mangalore, Manipal Academy of Higher Education, Manipal, India; 3 3Department of Neurology, Kasturba Medical College Mangalore, Manipal Academy of Higher Education, Manipal, India

**Keywords:** Health care, Medical research, Neurology

## Abstract

The Bedside Upper Limb Functional Evaluation Tool (BUFET) is a newly developed tool that assesses upper extremity functions, including proximal and distal movements such as gestures, gripping, and independent finger movements. This study aimed to evaluate the intra-rater and inter-rater reliability and predictive validity of the BUFET. Subjects ≥ 18 years of age and with a first episode of supratentorial stroke were included. The principal investigator, a physiotherapist, rated the subjects while performing the scale which was videotaped. To establish intra-rater reliability, the same rater re-scored the videos on two separate occasions, after 7 and 30 days. For inter-rater reliability, the principal investigator and 4 additional raters independently scored the patient based on the video. For predictive validity, the Wolf Motor Function Test (WMFT) was administered on the same day as BUFET, followed by administering the scales after 30 days. Intra- and inter-rater reliability was found to be excellent (ICC—0.992, 0.985). The reliability of all the components was > 0.89. The predictive validity was demonstrated by R^2^ values were 0.479 when compared with the WMFT scale. The study concluded that BUFET is an instrument with high intra-rater and inter-rater reliability with moderate predictive validity.

## Introduction

Reduced upper extremity (UE) function is one of the most common impairments after stroke and affects nearly 80% of patients in the acute phase and over 55% in the chronic phase, with about one-third experiencing difficulty in controlling fine movements^1^. Paresis, fractionated movement loss, abnormal tone, and somatosensory changes result from damage to the primary motor cortex, primary somatosensory cortex, subcortical structures, and corticospinal tracts^2^.

Such impairments result in functional limitations of the upper extremity, including proximal muscle weakness and deficits in multi-joint reaching and grasping. A significant loss of independent digit movements is observed, with finger extensors being most commonly affected, leading to loss of dexterity and an inability to manipulate objects. Compensatory strategies described for reaching and grasping include trunk flexion rather than elbow extension to reach for objects, forearm pronation, wrist flexion rather than neutral forearm position and wrist extension to orient the hand for grasping, and metacarpophalangeal joint flexion rather than proximal interphalangeal joint flexion to grasp objects^3,4^.

Adequate knowledge about the impairments in the early stages is necessary to determine appropriate interventions that would help reduce the disease’s impact. The selection of proper outcomes leads to a better understanding of the clinical condition. Given the heterogeneity of stroke etiology and symptoms, a battery of measures can be used to assess the functional status^5^. The Standardizing Measurement in Arm Rehabilitation Trials (SMART) Toolbox is a list of measures developed after a thorough systematic review and helps in the selection of outcomes to be used in post-stroke arm rehabilitation. The outcome measures include the Fugl-Meyer assessment (FMA), the Wolf Motor Function Test (WMFT), the Action Research Arm Test (ARAT), the Box and Block test, the Chedoke Arm and Hand Inventory, the Nine Hole Peg Test, the modified Rankin Scale, and the Canadian Occupational Performance Measure^6^. The most commonly recognized and applied outcome measure includes the Fugl-Meyer Assessment – Upper Extremity (FMA-UE) and WMFT^7^. The FMA-UE assesses the overall impairment of UE in the stroke population, including domains such as motor function, balance, sensory function, joint range of motion (ROM), and joint pain^8^. While the WMFT belongs to the activity domain of the International Classification of Functioning, Disability and Health (ICF), and consists of a series of UE functional tasks progressing from simple movements in proximal joint areas to complex movements in distal joint areas^9^. Several studies evaluating the psychometric properties of these measures demonstrated excellent reliability, validity, and responsiveness^10,11^. Studies conducted on kinematic assessment of UE, such as 2-D and 3-D motion analysis, focus on variables including movement speed, peak velocity, number of velocity peaks, path length ratio, trunk displacement, and shoulder and elbow joint angles. The lack of standardization of these tools and the evaluation of the psychometric properties of such measures limit their effectiveness as clinical assessment tools^12^.

Several kinematic studies and clinical outcomes can be used to measure the motor recovery of the UE post-stroke. The results derived from both impairment and functional assessments assist the clinician with developing treatment plans and aid in evaluating the utility of a particular treatment. Limitations of these measures include time constraints, the need for specialized equipment, and the requirement of trained professionals^5^.

The Bedside Upper Limb Functional Evaluation Tool (BUFET) is a newly developed tool that can be administered bedside with ease, accuracy, minimal time consumption, and less exhaustion. The BUFET has 12 components, of which three components assess the proximal glenohumeral joint along with the elbow and radioulnar joint, one assesses coordinated joint movements, and the rest of the components assess hand function, including day-to-day movement gestures, grasping activities, and rhythmic wrist and finger movements. The scale was developed after a thorough review of published literature. The content and construct validity of the scale were established following the mini-Delphi consensus method involving six experts from the field of neurological clinical practice. The items of the scale were selected based on clinical relevance, feasibility, and expert opinion, with consideration of task-specific and impairment-specific characteristics. For concurrent validity, BUFET and WMFT were administered to subjects, and the scores of both tests were compared; the results showed a high correlation coefficient (*r* = 0.937)^13^. The objective of the present study was to further assess the psychometric properties of the BUFET. The intra-rater reliability, inter-rater reliability using multiple raters, and predictive validity of the scale were evaluated to establish the scale as a valid and reliable tool that can be used for clinical and research purposes.

## Methods

### Study setting and ethical approval

The study sample was recruited through convenience sampling among post-stroke subjects admitted to the Kasturba Medical College Mangalore from June 2024 to March 2025. Ethical approval was obtained from the Institutional Ethics Committee of Kasturba Medical College Mangalore (IEC KMC MLR 12/2023/482). The study was registered under the Clinical Trials Registry- India (CTRI No. – CTRI/2024/06/069259). All the subjects provided written informed consent. The study was performed in accordance with the Declaration of Helsinki.

## Participants

Subjects were included if they met the following inclusion criteria: (1) age between 18 and 80 years (2) primary ischemic/hemorrhagic stroke with upper limb involvement (3) stroke duration of < 3 months. Subjects were excluded if they (1) had any other neurological conditions (2) had severe cognitive deficits (St. Louis University Mental Status [SLUMS] score ≤ 26) (3) had any pre-existing upper limb musculoskeletal conditions which could have affected the testing method.

## BUFET scale

BUFET consists of 12 components, each with a score of 0–4, where 0 denotes that the subject is unable to perform the movement, and 4 denotes that the subject is able to complete the task or action without any deviation from the normal pattern^13^. Further details about the scale and the scoring system are available from 10.17605/OSF.IO/UFHK5.

## WMFT

The WMFT consists of 15 items which are time based rated on a six-point functional ability scale, assessing effort, smoothness, and overall quality. It takes 30–35 min to complete the evaluation. WMFT exhibits excellent test-retest reliability (*r* = 0.95) and strong inter-rater (ICC = 0.93) and intra-rater (ICC = 0.97) reliability^9,14^.

## Procedure

A total of 5 raters were involved in the scoring of the participants, including the principal investigator (PI). The subjects were rated by the PI on the first day of referral, and simultaneously, a video recording of the participants performing the components was obtained. This recording was later used by the four independent raters to score the subjects for assessing inter-rater reliability. For intra-rater reliability, the PI re-scored the same recordings at day 7 and day 30 after the initial assessment.

### For intra-rater reliability

The PI, blinded to the previous scores, re-assessed the video recordings twice: at 7 days and 30 days following the initial assessment. All three scores were used to establish intra-rater reliability.

## For inter-rater reliability

Four other raters (postgraduate students who were proficient in administering the scale) scored each study participant based on the video recording of him/her performing the activities. The scores of the 4 raters and the PI were used to establish the inter-rater reliability.

## For predictive validity

To establish the predictive validity, the WMFT scale, a core outcome measure considered as a gold standard in the present study, was administered on the same day as BUFET, i.e., on the first day of referral. Both the scales were again administered to the subject after one month of duration. The initial score as well as the post-one-month score of the BUFET and WMFT scale was compared. The baseline scores were collected by the PI, and follow-up values were collected by another assessor.

### Data analysis

The data collected was subjected to analysis using Jamovi version 2.6.44 for Windows. The normality of distribution was calculated by Shapiro Wilk Test. Intra-rater reliability was established from three different ratings of the same rater and inter-rater reliability was from a rating of multiple raters using the two-way mixed effects model Intraclass Correlation Coefficient [ ICC (3,k) ]. Predictive Validity was estimated by deriving a correlation coefficient followed by linear regression. Statistical significance was set at *p* < 0.05, and all estimates were reported with 95% confidence intervals.

## Results

A total of 34 participants were recruited in this psychometric study. The demographic and clinical characteristics are mentioned in the table below (Table 1).

**Table 1:** Demographics and clinical characteristics.

Variables	Characteristics	Mean ± SD	*n* (%)
Age (Years)	–	59.8 ± 11.0	34(100)
Gender	Male	–	25(73.52)
	Female	–	9(26.47)
Lesion type	Infarct	–	27(79)
	Hemorrhage	–	7(21)
Side of lesion	Left	–	13(38)
	Right	–	21(62)
Hand dominance	Left	–	2(5.8)
	Right	–	32(94.1)
SLUMS score	–	27.4 ± 1.7	34(100)
BRS	–	3.5 ± 1.54	34(100)

SD, Standard Deviation; SLUMS, St. Louis University Mental Status Examination; BRS, Brunnstrom Recovery Stage.

### Intra-rater reliability

**Fig. 1:**
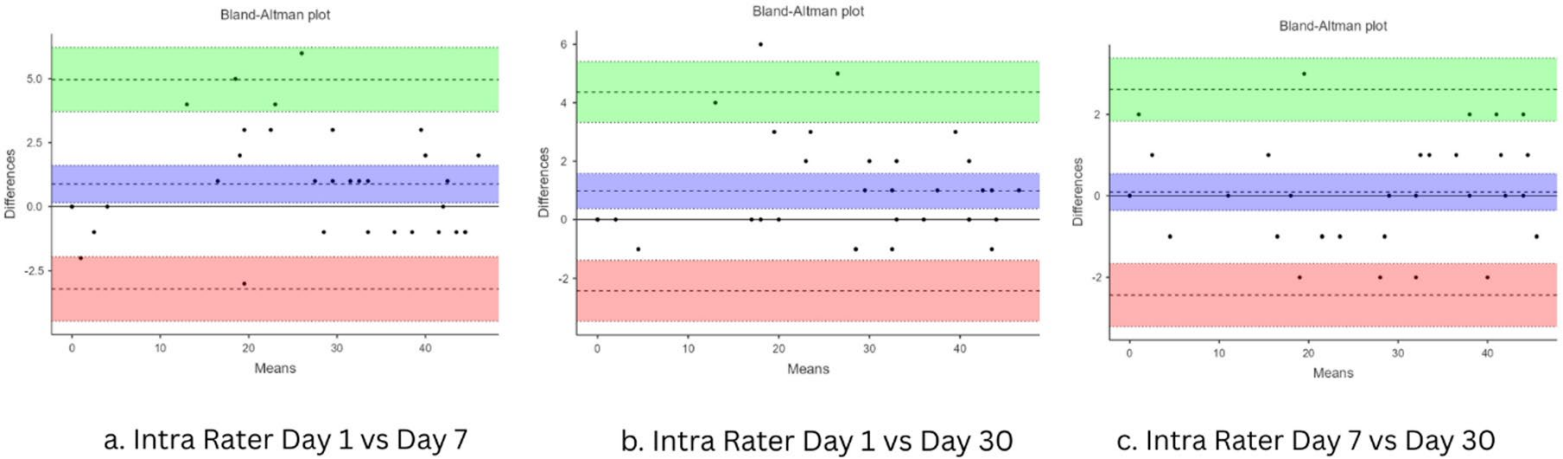
Bland–Altman plot showing the intra-rater agreement of Rater 1 across sessions (Day 1, Day 7, and Day 30).

The intra-rater reliability was found to be excellent, with an ICC agreement of 0.992 (95% CI: 0.986–0.999), indicating high consistency across repeated assessments by the same rater. The component-wise intra-rater agreement values ranged from 0.88 to 0.98, indicating good to excellent reliability across the components of the tool (Table 2) Agreement was assessed using Bland–Altman analysis, and the corresponding plots are presented in the figure below (Fig. 1).

**Table 2:** Intra-rater agreement of 12 components.

Component	Agreement
Salute	0.98
Hold the Nose	0.97
Hand waving	0.92
Clockwise and anticlockwise stirring action of wrist	0.93
Point the index finger upwards with wrist in extension	0.93
Grip the examiner’s fingers	0.93
Oppose and maintain the contact of thumb and little finger	0.91
Hold the examiner’s finger using the thumb and index finger	0.93
Snapping action of the fingers	0.92
Gesture the number 3 using middle, ring, and little finger	0.92
Gesture a scissoring action using the index and middle finger	0.95
Finger tapping	0.89

### Inter-rater reliability

**Fig. 2:**
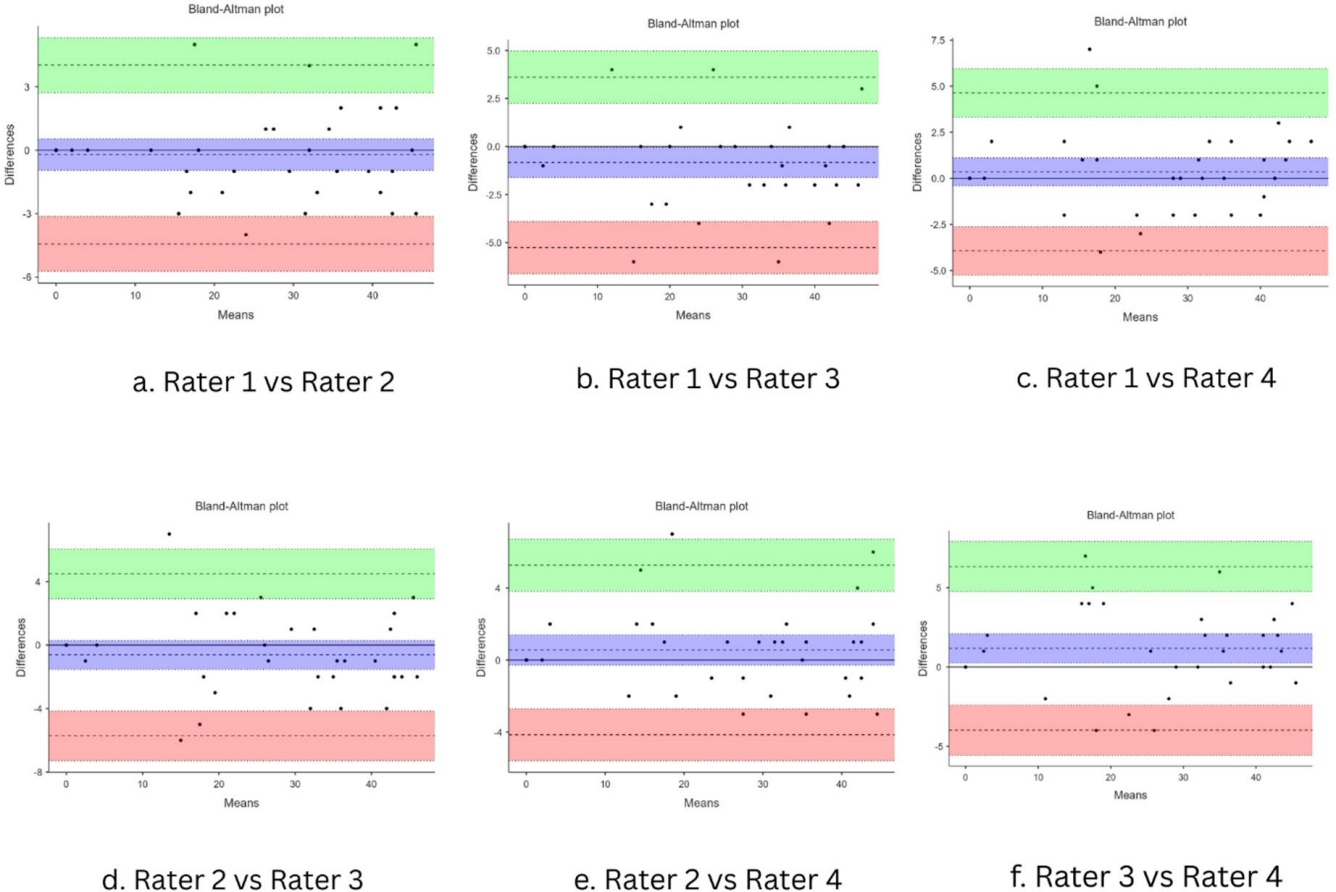
Bland-Altman plots showing agreement between different raters.

The scale’s inter-rater reliability was reported to be excellent with an ICC of 0.985 (95% CI: 0.976–0.996). The component-wise inter-rater agreement values ranged from 0.87 to 0.95 indicating good to excellent reliability across the components of the tool (Table 3). Agreement was assessed using Bland–Altman analysis, and the corresponding plots are presented in the figure below (Fig. 2).

**Table 3:** Inter-rater agreement values of 12 components:

Component	Agreement
Salute	0.95
Hold the Nose	0.94
Hand waving	0.88
Clockwise and anticlockwise stirring action of wrist	0.91
Point the index finger upwards with wrist in the extension	0.89
Grip the examiner’s fingers	0.92
Oppose and maintain the contact of thumb and little finger	0.88
Hold the examiner’s finger using the thumb and index finger	0.92
Snapping action of the fingers	0.87
Gesture the number 3 using middle, ring, and little finger	0.90
Gesture a scissoring action using the index and middle finger	0.89
Finger tapping	0.88

### Predictive validity

Linear regression was performed to evaluate the predictive validity of the BUFET by comparing the scores with changes in upper limb function as measured by WMFT. The R-value was 0.692, R^2^ = 0.479, *p* < 0.001, indicating that 47.9% of the variance in WMFT change scores could be explained by changes in BUFET scores. BUFET change score significantly predicted WMFT change (B = 1.42, 95% CI: 0.91–1.93, *p* < 0.001). Even when the regression model was adjusted for probable confounders such as age, stage of recovery, side, and severity of lesion, BUFET was still found to be a highly significant predictor, R^2^ = 0.524, with *p* < 0.05. The regression model upon bootstrapping returned a coefficient 0.337 (95% CI 0.185 to 0.518, *p* = 0.003), suggesting statistically significant association between the study variables.

### Correlation of BUFET and WMFT change

**Fig. 3:**
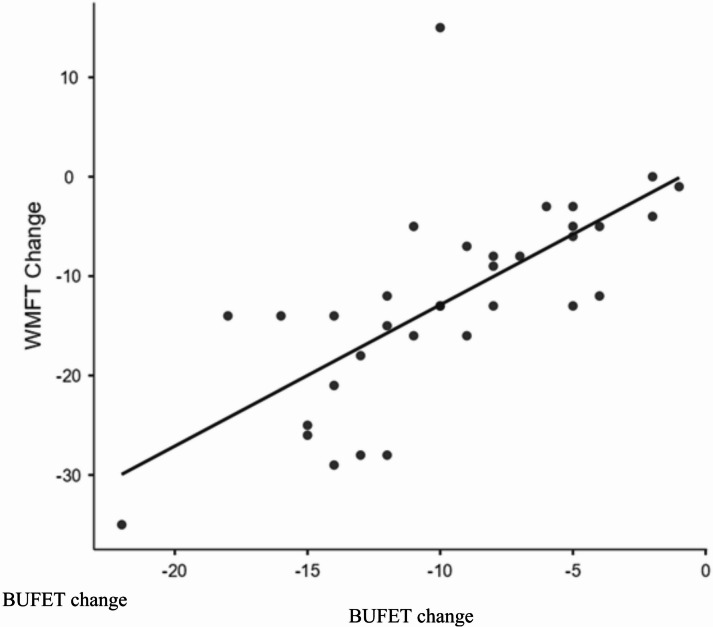
Scatter plot denoting the changes of BUFET and WMFT over time.

A scatter plot was generated to highlight the relationship between changes in BUFET and WMFT scores. The plot demonstrated a clear positive linear trend, indicating that participants who showed greater improvements on the BUFET also demonstrated greater improvements on the WMFT (Fig. 3).

### Concurrent validity

As an additional finding from existing data, the concurrent validity of the scale was assessed. The BUFET score on day 1 was correlated with the WFMT score on the same day using Spearman’s rank correlation coefficient. A strong positive correlation was found between the two measures (Spearman’s ρ = 0.957, *p* < 0.001), indicating excellent concurrent validity of the BUFET in relation to the WFMT. The concurrent validity observed between the BUFET and the WMFT in this study is comparable to values reported in an earlier study (*r* = 0.937; *p* < 0.001)^13^, indicating consistency of results across different samples. While the two coefficients are not the same statistic, both show a positive correlation supporting the conclusion that BUFET has excellent concurrent validity with WMFT.

## Discussion

Current assessment tools for the evaluation of motor impairments of the shoulder, elbow, wrist, and hand require specialized equipment, trained professionals and considerable time to administer. The BUFET was developed as a simple, resource-efficient and time-efficient tool that can be completed in under 10 min. The scale was developed and validated using the mini-Delphi method^13^. Considering the novelty of the tool, a rigorous assessment of psychometric properties is essential to establish the scale for implementation for both clinical and research purposes.

In the present study, the intra-rater and inter-rater reliability of the BUFET was assessed, along with a component-wise analysis. In a study conducted by Morris et al., the inter-rater reliability of WMFT was shown to have agreement values of 0.88, while the agreement for individual test items was in the range of 0.50–0.93. An agreement value of less than 0.75 was observed in about 9 items. Meanwhile, the test-retest agreement value was reported to be 0.95^10^. Studies on modified versions of WMFT such as graded WFMT reported a high inter-rater reliability with ICC = 0.98^14^. Other frequently used UE outcome measures such as FMA-UE demonstrated an ICC range from 0.93 to 1.00 for inter-rater reliability and ICC = 0.97 for test-retest reliability^11^, while ARAT showed an ICC = 0.98 for inter-rater reliability^15^. The current study also demonstrated high values of reliability within the rater and across raters, with ICC values of 0.992 and 0.985 respectively, the component-wise agreement for both intra-rater and inter-rater was found to be significant for all 12 components, ranging between 0.89 and 0.98 agreement within rater and 0.87–0.95 across multiple raters.

Predictive validity was examined to evaluate whether the BUFET could accurately predict changes in upper limb function, as measured by the WMFT. Linear regression analysis showed a statistically significant relationship, indicating that nearly half the variance (47.9%) in WMFT scores could be explained by changes in BUFET scores. The R^2^ values of 0.479 demonstrate good predictive validity and suggest that the scale can be used to anticipate functional upper limb improvements following stroke^16^. Studies conducted for assessing the predictive validity of other UE measures such as STREAM-UE reported *R* = 0.72 when compared with the FMA-UE, stating that the predictive power of the scale was satisfactory^17^. Similar results were demonstrated in this study where the R-value was 0.69 when BUFET was compared to WMFT, suggesting that BUFET has a moderate predictive power when compared to other standard UE measures.

The WMFT consists of 17 tasks including 15 functional tasks and 2 strength tasks. The evaluation is based on a 6-point functional ability scale (WMFT-FAS) and time of performance (WMFT-TIME). It is a unidimensional tool, and the items consist of a progression of tasks from simple to complex tasks and fine motor activities^9,18,19^. Out of the 15 functional tasks, 40% of the tasks focus on proximal joint movements while the rest of the tasks assess the distal joints and hand functions such as grasping objects. On the contrary, the BUFET has a total of 12 components, out of which 3 components assess proximal joint function (25%), 3 tasks of gripping, 4 distal joint movements i.e., gestures, and 1 task assessing independent digit movement^13^. Hence, the scale is an extensive measure of UE functions including proximal inter-joint movement, fine motor activities, dexterity, and coordination of the distal joints. Previous studies have established that gestures and grasping are associated with deviations in joint rotation patterns, altered hand orientations, and disrupted inter-joint coordination during complex hand gestures and grasp-twist movements^20^. The BUFET broadly assesses different motor functions of the UE and is a unidimensional tool. Due to the similarity of assessment of motor function, WMFT was chosen as a comparator in this study. The WMFT has well-established psychometric properties, including high reliability, validity, and responsiveness in post-stroke populations. Consequently, BUFET could lead to results comparable to WMFT to quantify the degree of motor impairment and predict the anticipated stage of recovery. The results can guide clinical decision-making, the efficacy of interventions, and monitor patient’s progress.

The present study is the first comprehensive evaluation of the psychometric properties, reliability, and validity of the BUFET. Due to the high reliability and validity found in the study, the scale can be used alongside other recommended measures such as the SMART toolbox, which provides a list of outcomes important to stroke subjects, their caregivers, and clinicians^6^. The ICF categorizes WMFT under the activity domain as a scale consisting of various UE movements and functional tasks^21^. Multiple studies have demonstrated that the scale has good psychometric properties^9,10,14,19^. The items of BUFET consisting of assessment of the proximal aspect of UE, gestures, and independent digit movements are comparable to WMFT, suggesting that this scale has considerable activity domain components in addition to selective body structure and function components of ICF.

The results of the outcome are satisfactory due to several reasons. First, the study included people with acute stroke with different Brunnstrom stages of recovery, resulting in variability of test scores, which increases the generalizability of the study. Second, the standardized, equipment-free methodology and well-trained raters may have helped to minimize any measurement errors. And, third, approximately 10 min is required to administer the scale, which would have allowed the subjects to perform the movements optimally.

### Limitations

The sample size of the current study was relatively small, due to which the specificity and sensitivity of the scale could not be assessed. In addition to the above, a single-center design, homogeneous sample and absence of clinical applicability of potential cut-off scores may limit the generalizability of the study findings. The BUFET was correlated with the WMFT, which is a function-based scale of the UE. Other commonly used scales with high degrees of psychometric properties, such as the FMA-UE or the ARAT were not correlated in the present study.

## Conclusion

This study investigated the psychometric properties of the BUFET, which was developed to measure upper limb function in individuals following a stroke. The results demonstrated that the scale has excellent reliability and predictive validity, suggesting that it is applicable in both clinical practice and research settings. The BUFET effectively identifies functional and physiological impairments, progress in upper limb recovery, and moderately predicts changes in motor capacity, offering a practical, evidence-based option for physiotherapists and rehabilitation professionals. Future studies with larger and more diverse populations are recommended to further establish its responsiveness and applicability.

## Data Availability

Data supporting the findings of the study are available in doi: https://doi.org/10.17605/OSF.IO/BPV8X.
